# Spike timing in rat somatosensory cortex contributes to behavior

**DOI:** 10.1186/1471-2202-14-S1-P109

**Published:** 2013-07-08

**Authors:** Alberto Mazzoni, Zuo Yanfang, Giuseppe Notaro, Stefano Panzeri, Mathew E Diamond

**Affiliations:** 1 Center for Neuroscience and Cognitive Systems, Istituto Italiano di Tecnologia, Rovereto (TN), 38068 Italy; 2 Cognitive Neuroscience Sector, International School for Advanced Studies, Trieste, 34136, Italy; 3 Max Planck Institute for Biological Cybernetics, Tübingen, 72076, Germany; 4 Institute of Neuroscience and Psychology, University of Glasgow, Glasgow G12 8QB, UK

## 

The precise spike timing of sensory neurons carries more information about stimuli than the number of spikes counted over longer time windows [1-3]. However, the behavioral relevance of this extra information remains to be assessed. To investigate whether precise timing contributes to behavior, we considered spike trains recorded extracellularly in the rat somatosensory cortex during a texture discrimination task [4,5].

We analyzed the spike trains following the contact of the whiskers with the texture and we measured the information carried about texture by post-touch spike times and spike counts. This analysis is technical challenging because it is difficult to quantify the information content of precise patterns of spike that extend over relatively long time windows with the relatively little number of trials available in behavioral experiments. To address this challenge, we reduced the dimensionality of the response space by decomposing single trial spike train densities into Principal Components (PCs), selecting the most informative PC as the "temporal template" to interpret neural responses, and finally measuring the information about the presented texture carried by the projection of each trial relative to this template. We compared the so -measured spike timing information with the information carried by spike counts and we found that spike times carried more than 60% more texture information than spike counts.

Next, we reasoned that if a neuronal coding mechanism contributes to behavior, then the code would be found to transmit less stimulus information in error trials, i.e. trials in which the rat made the wrong choice. We found that the texture information carried both by spike counts and spike patterns decreased significantly when we included error trials in the analysis, but the effect was much more pronounced for patterns (30% information decrease) than for counts (~15% information decrease). These differences between spike pattern and spike count codes hold for both primary and secondary somatosensory cortex, suggesting that spike timing could contribute to behavior at different stages of the sensory processing.

Taken together, these results indicate that in the somatosensory cortex the time at which spikes occur, not just the number of spikes, makes a crucial contribution to tactile perception.
